# Influence of Crude Oil Production Chemicals on the Stability of O/A Emulsions and on Flocculant Efficiency

**DOI:** 10.1021/acsomega.6c05554

**Published:** 2026-10-13

**Authors:** Paulo C. S. Rocha, José V. L. Silva, Rita C. P. Nunes, Elizabete F. Lucas

**Affiliations:** † 28125 Federal University of Rio de Janeiro, Macromolecules Institute, Rua Moniz Aragão, 360, block 8G/CT2, 21941594 Rio de Janeiro, RJ, Brazil; ‡ Federal University of Rio de Janeiro, Metallurgical and Materials Engineering Program/COPPE, Av. Horácio Macedo, 2030, block F, 21941598 Rio de Janeiro, RJ, Brazil

## Abstract

The water produced during oil extraction contains oil emulsified. Therefore, before being reinjected or discarded, this produced water must comply with the oil in water concentration limits established by Brazil’s environmental regulatory authority, CONAMA. These limits correspond to a monthly average of 29 mg/L and a maximum daily value of 42 mg/L. Treatment of the emulsified fraction, one the most resistant to separation, is commonly carried out flocculation with water-soluble polymers. In this work, synthetic oily waters using heavy oil were prepared, and two types of cationic polymeric flocculants were evaluated, both individually and in combination with a commercial demulsifier and an anionic SPAM polymer, which are commonly used in oil production operations. For this purpose, flocculation tests (jar tests) were performed on oil in water emulsions prepared with different oil concentrations. The results showed that the demulsifier alone reduced the oil removal efficiency of the flocculants. Conversely, the presence of SPAM alone further reduced the performance of the flocculants, at both oil concentrations tested. When used together, these additives almost completely suppressed the action of the flocculants. Particle size analyses confirmed that the most efficient flocculant promoted the formation of larger aggregates, while the presence of the demulsifier and SPAM limited this formation. Interfacial tension measurements showed that the flocculants’ action was directly related to the availability of interfacial area, being impaired when this interface was previously occupied by demulsifier and SPAM.

## Graphical abstract

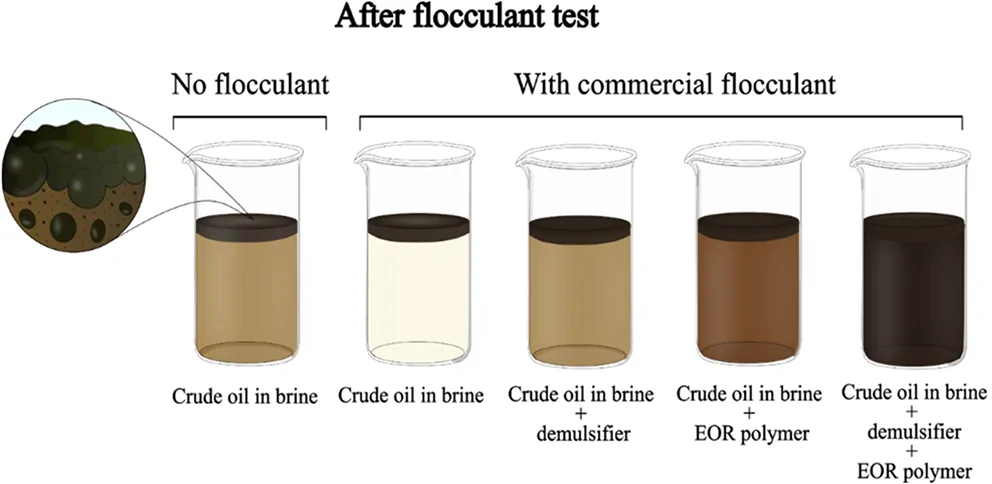

## 1 Introduction

The growing global demand for energy has intensified oil exploration and production, a process that inevitably results in the generation of large volumes of produced water, also known as oily water, a complex and challenging effluent from environmental and technological standpoints.^1,2^ This water, originating from the reservoir itself (connate water) or injected for secondary and tertiary recovery purposes, has a highly variable composition. The produced water can contain salts, total dissolved solids (TDS), oils and greases, benzene, toluene, ethylbenzene and xylenes (BTEX), polycyclic aromatic hydrocarbons (PAHs), organic acids, phenol, inorganic compounds (silicas, scale-forming salts, metals, radioactive materials, among others), organic compounds (light or heavy crude oil and gases), as well as various microorganisms (bacteria, fungi and algae).^3^ Various chemicals added during the oil production process, aimed at solving or preventing operational problems, can also be present in produced water, such as scale inhibitors, corrosion inhibitors, hydrate formation inhibitors, biocides, demulsifiers, antifoaming agents, and polymers used in enhanced oil recovery (EOR) techniques.^4–8^ The pH is also considered a problem, being responsible for the corrosion of equipment, as is the presence of chloride ions. These ions in excess, in addition to the corrosive process, can cause scaling, since they increase the hardness of the water.^9,10^

Both in onshore and offshore production, the volume of produced water can be very large. Indeed, it is considered the main waste product generated by the petroleum industry. The water/oil ratio (WOR) is among the metrics used to define the characteristics and productive life stages of reservoirs. It is estimated that this ratio is, on average, 3:1, meaning that 3 barrels of water are generated for every barrel of oil produced, making the treatment and disposal of effluents one of the biggest challenges for the oil sector.^1,2^ In offshore fields, produced water can be discharged near the production field or reinjected into the reservoir. However, the effluents must meet certain quality standards to avoid environmental impact (marine pollution, contamination of water bodies and aquifers, soil salinization, damage to fauna and flora, harm to human health) or damage to oil production itself, in the case of reinjection.^6,11–14^

Oil can be present in oily water in dispersed, dissolved, suspended, and emulsified forms. The presence of stable oil-in-water (O/W) emulsions makes the treatment of produced water particularly challenging, since these emulsions exhibit high resistance to separation by conventional methods such as flotation, sedimentation, and/or the use of membranes.^15,16^ The stability of these emulsions is associated with factors such as droplet size, interfacial tension, the presence of natural surfactants (asphaltenes, resins) and chemical additives, which form viscoelastic films around the droplets, hindering their coalescence.^17–23^

Among the chemical technologies applied for the treatment of produced water, flocculation with water-soluble polymers has proven to be one of the most effective, especially for the removal of colloidal particles and emulsified oil.^24–29^ Flocculation is a complex process whose efficiency depends on several factors, such as the nature of the flocculant and its dosage; the method of application; the chemical environment; the hydrodynamic system; the molecular state; particle size; pH and salinity.^30,31^ Flocculants act through various mechanisms, such as the formation of polymer bridges, charge neutralization, and dual flocculation, and their efficiency is affected by variables such as molar mass, charge density, polymer chain conformation, and the physicochemical conditions of the medium.^32–38^

The residual presence of certain additives used in oil production, such as demulsifiers and anionic polymers employed in enhanced oil recovery techniques (for example, partially hydrolyzed polyacrylamide) can significantly reduce the performance of flocculants.^9–40^ These additives can alter the interfacial tension and Zeta potential of the system, hinder the adsorption of the flocculant onto the target particles, or modify the conformation of the polymer in solution, negatively impacting the formation and stability of the flocs.^41–43^

Despite the importance of the topic, the combined effects of these additives on the performance of polymeric flocculants are still poorly explored and poorly understood, especially in systems representative of produced water containing heavy oil, high salinity, and stable emulsions. Therefore, it is necessary to systematically investigate these interactions, correlating droplet size and interfacial tension with operational and performance parameters of flocculants, such as total oil content (TOG) reduction. Given the complexity arising from the composition of produced water and the limitations associated with conventional treatments, it is essential to deepen investigations into the effects of residual chemical additives on the performance of the polymeric flocculants applied in oil–water separation. Understanding these interactions is fundamental not only to optimize treatment processes, but also to develop more efficient and environmentally compatible technological solutions that meet the requirements of the oil industry. In this context, this work presents a systematic and well-founded approach to evaluate the individual and combined impacts of an anionic polymer used in enhanced oil recovery (EOR) and a commercial demulsifier on the performance of two commercial cationic polymeric flocculants. The systems were evaluated using the jar test method, with determination of the TOG in the water before and after treatment, as well as by determining particle sizes and interfacial tension levels.

## 2 Materials and Methods

### 2.1 Materials

A heavy crude oil sample (°API 13.25, density 0.9766 g/cm^3^, water content ∼2.8%w/t asphaltenes content 13.6% w/t and viscosity 1 Pa·s at 60 °C), two commercial flocculants (a mixture of formaldehyde and glyoxal with cationic polycondensate in aqueous medium, designated flocculant A, and a mixture of tetrakis hydroxymethylphosphonic acid with cationic polycondensate in aqueous medium, called flocculant B), the commercial thickener FLOPAAM AN125 (copolymer of acrylamide and acrylamido propyl sulfonated acid, molar mass: 8,000,000 Da and 25 mol % of sulfonate monomer), named SPAM, and a commercial demulsifier based on poly­(propylene oxide-*b*-ethylene oxide) were supplied by Equinor (Brazil). Acetic acid (CH_3_COOH) (ACS), sodium bicarbonate (NaHCO_3_) (ACS), strontium chloride hexahydrate (SrCl_2_.6H_2_O) (ACS), potassium chloride (KCl) (ACS), sodium chloride (NaCl) (ACS) and sodium hydroxide (NaOH) (PA) were acquired from Isofar (Duque de Caxias, Brazil). Barium chloride dihydrate (BaCl_2_.2H_2_O) (PA), magnesium chloride hexahydrate (MgCl_2_.6H_2_O) (PA), calcium chloride dihydrate (CaCl_2_.2H_2_O) (PA), hydrochloric acid (HCl) (PA) and *n*-hexane (PA) were purchased from Labsynth (Diadema, Brazil). Silicon dioxide (SiO_2_) (Emprove Ph Eur brand) was acquired from Merck Brasil (Rio de Janeiro, Brazil). Distilled water was used to prepare the emulsions.

### 2.2 Characterization of Production Chemicals and Flocculants

#### 2.2.1 Determination of Specific Charge Density (SCD)

The specific charge densities of the demulsifier, SPAM, and flocculants A and B samples were determined using the PCD-06 equipment (Mütek—BTG), based on the principle of electrokinetic shear, generating an electric field whose intensity was recorded as flux potential (mV). For the quantification of SCD, solutions were prepared at 100 ppm and, in each assay, 5 mL was titrated with a polyelectrolyte of opposite charge until the flow potential reached 0 mV, characterizing the equivalence point. The analyses, performed in duplicate, allowed us to obtain the initial flow potential, the ionic demand (volume of titrant consumed until neutralization), and the charge density of the sample. The specific charge density per mass of the sample was calculated using eq 1. Where: meq = mass charge density (eq/g); *V* = volume of titrant consumed (*L*); *c* = concentration of the titrant (eq/L) and *m* = sample solids or active substance (g).

1 $$meq=\frac{V\times c}{m}$$

### 2.3 Brine Preparation

The salts were gradually dissolved in deionized water using a stirring plate at room temperature, at the following concentrations: sodium chloride (80.8 g/L); calcium chloride dihydrate (8.73 g/L); magnesium chloride hexahydrate (4.34 g/L); potassium chloride (0.81 g/L); strontium chloride hexahydrate (0.72 g/L); sodium bicarbonate (0.2 g/L); barium chloride dihydrate (0.035 g/L); and silicon dioxide (0.025 g/L). After complete dissolution, 290 μL acetic acid was added. Finally, the pH of the solution was adjusted to 7 using sodium hydroxide or a hydrochloric acid solution. For tests involving the addition of 450 ppm of SPAM, it was previously weighed and dissolved in synthetic brine under magnetic stirring at 80 °C for 90 min.

### 2.4 Oily Water Preparation

The oil was heated to 80 °C and weighed into a beaker. Then brine, also previously heated to 80 °C, was added to the same beaker. The phases were stirred at 15,000 rpm using a Polytron (PT 10–35 GT) for 9 min at room temperature. The emulsion was then transferred to a 2000 mL separatory funnel and allowed to stand for 1 h. After transferring the oily water to the funnel and the 1 h resting period, it was observed that a large amount of oil remained retained to the funnel walls. Following the collection of aliquots, preliminary tests revealed significant differences in TOG values among the aliquots analyzed using the Jar Test method.

This behavior can be attributed to the nonuniform distribution of the crude oil within the brine, which prevented the collection of samples with similar concentrations for the flocculation assay. Consequently, after the funnel had stood for 1 h, the oily water was transferred to a second funnel. In the funnel receiving the freshly prepared oily water, the variation in oil concentration throughout the system was evident. In contrast, the oily water drained into the second funnel exhibited a more uniform color, indicating a more regular distribution of the crude oil within the brine.

After this procedure, the emulsions were collected in the following order: (1) 45 mL into a graduated cylinder and (2) 200 mL into a beaker, alternately, until five graduated cylinders and five beakers were filled. The samples from the graduated cylinders were analyzed as the blank from their respective beakers This procedure was also carried out with the aim of keeping all aliquots as uniform as possible.

### 2.5 Flocculation Test (Jar Test)

The samples contained in the beakers were subjected to the jar test using The Compact Laboratory Mixer, remaining under agitation at 388 rpm for 2 min and then under agitation at 20 rpm for 10 min, and at rest for 20 min. At the end of the test, a 45 mL aliquot was removed from each beaker and placed in graduated cylinders, and 5 mL of *n*-hexane was added, followed by manual agitation for 1 min. For complete phase separation, the liquid was poured into graduated cylindrical tubes, which were centrifuged for 5 min at 1500 rpm. At the end of the centrifugation, complete separation of the organic phase from the aqueous phase occurred. Then, 1 mL of the organic phase was removed from each sample using a micropipette. This volume was diluted (1000× with the extraction solvent) to allow for TOG measurements using a benchtop fluorimeter (TD 3100). All analyses were performed in duplicate.

### 2.6 Flocculation Test (Jar Test) with and without the Use of Chemicals and SPAM

Flocculation tests were performed with and without 450 ppm of SPAM in brine using flocculant A or B, with and without the addition of a demulsifier. Efficiencies were reported in terms of percentage reduction in TOG compared to the TOG before the jar testing procedure.

#### 2.6.1 Only Demulsifier with and without SPAM

The demulsifier was added at concentrations of 0, 50, 175, and 350 ppm, allowing the evaluation of its performance both alone and in combination with SPAM. The demulsifier was added to the beaker containing oily water before the flocculation tests.

#### 2.6.2 Only Flocculant with and without SPAM

Tests were conducted to evaluate the efficiency of flocculants A and B with and without SPAM. Flocculants A and B at concentrations of 0, 50, 100, 200, 300, 400, and 500 ppm and were added to the beaker containing oily water before the flocculation tests.

#### 2.6.3 Demulsifier + Flocculant with and without SPAM

Tests were conducted to evaluate the influence of the demulsifier, alone and in combination with SPAM, on the performance of flocculants A and B. For this purpose, demulsifiers were used at concentrations of 0, 50, 175, and 350 ppm, and flocculants at concentrations of 0, 50, 100, 200, 300, 400, and 500 ppm. The chemicals were included first by adding flocculant A or B, followed by the demulsifier to the beaker containing AO before the flocculation test. All tests were performed in duplicate, and the results are expressed as the average of the values obtained.

Additive concentrations may vary depending on the oil field. However, in this study, concentrations were based on the dosages used in a Brazilian heavy-oil field.

### 2.7 Particle Size

The size and particle size distribution of the oil/water emulsions were determined by laser diffraction using a Mastersizer 3000 (Malvern) coupled to the Hydro EV system at 1500 rpm. Approximately 150 mL of the emulsion was added to the disperser. After an obscurity index in the range of 10–20% was reached, three consecutive measurements were performed on each sample. The results were expressed as D10, D50, and D90, meaning that 10%, 50%, and 90% of the particles were below this size, respectively. The analyses were performed in duplicate.

### 2.8 Interfacial Tension

The interfacial tension measurements were performed using a pendent drop tensiometer (Tracker). For analyses without chemical. The brine and oil were heated separately for 30 min at 80 °C and then 500 μL of oil was added to the equipment syringe and the brine was placed in a quartz cuvette. The drop volume used was 20 μL, the total analysis time was 1 h and the temperature was 60 °C. Since the analyses were performed at temperature above ambient, the measurements were made in function of the droplet surface area, to reduce the analysis error. For the analyses containing chemicals, the oil sample was heated in an oven for 30 min at 80 °C, and then the chemical was added and homogenized manually for 1 min. The mixture was left resting for at least 12 h. Subsequently, the brine and the sample containing the chemical were heated separately for 30 min at 80 °C, before starting the analysis. The equipment conditions were the same used for the samples without chemicals.

## 3 Results and Discussion

### 3.1 Determination of Specific Charge Density (SCD)

Determining the specific charge density (SCD) of the samples is a fundamental step in understanding the electrokinetic behavior of the analyzed compounds, particularly in colloidal systems where surface interactions play a decisive role in emulsion stability or instability. This analysis allows for the quantification of the net charge present per unit mass, enabling an assessment of the electrostatic interaction capacity of the various agents employed. The results obtained, including flow potential (mV), ionic demand (mL), and volumetric charge density (meq/L), as well as the values obtained using eq 1 mass charge density (meq/g), are presented in Table 1.

**Table 1:** Charge Density of the Materials That Make Up the Flocculation System

Materials	Flow potential (*M*_v_)	Ionic demand (mL)	Volumetric charge density (meq/L)	Mass charge density (meq/g)
Flocculant A	890.5 ± 11.5	0.195 ± 0.008	391 ± 1.50	0.39 ± 0.014
Flocculant B	787.0 ± 55.0	0.110 ± 0.000	223 ± 0.20	0.22 ± 0.002
Oily water	–1286.0 ± 40,0	0.078 ± 0.005	–153 ± 0.90	–0.15 ± 0.010
Demulsifier	–832.0 ± 76.5	0.088 ± 0.008	–175 ± 1.56	–0.17 ± 0.016
SPAM	–2503.0 ± 1.0	2.959 ± 0.167	–5,740 ± 30.45	–5.91 ± 0.334

The SCD values were determined to aid in the discussion of flocculant behaviors, with and without the presence of SPAM and/or demulsifier. Table 1 shows the charge density results, confirming the cationic nature of flocculants A and B, classified as cationic polycondensates by the manufacturer. Both presented low mass charge densities, with values of 0.39 ± 0.014 mequiv/g (flocculant A) and 0.22 ± 0.002 mequiv/g (flocculant B), indicating a limited amount of ionizable cationic groups available in the polymeric structure in solution, especially in flocculant B. The other materials analyzed showed negative charges. Oily water (−0.15 mequiv/g) and the demulsifier (−0.17 mequiv/g) exhibited similar values. SPAM stood out for presenting the highest negative charge density (−5.91 ± 0.334 mequiv/g), attributed to the presence of functional groups such as carboxyl and sulfonates.

### 3.2 Oily Water with and without SPAM

Samples of heavy, extra-heavy, and asphaltic crude oil present various handling challenges due to their high viscosity and tendency to adhere to various types of surfaces. Consequently, it was necessary to develop a methodology to ensure the preparation of oily water (with a salt content of 90,000 ppm) containing 1000 ppm of oil relatively well distributed within the brine that is, without exhibiting a significant oil concentration gradient inside the storage vessel. This is because, typically, enough oily water is prepared to analyze up to six samples using the Jar test apparatus; therefore, the samples must exhibit characteristics that are as similar as possible.

To achieve the desired concentration, it was necessary to adjust the concentration of oil mixed with the brine, progressively increasing it until values closer to the 1,000 ppm oil concentration in the final oily water were obtained (Table 2).

**Table 2:** TOG Determined by Fluorimetry

Concentration of oil mixed with brine (ppm)	TOG (ppm)
1000	27.35 ± 5.85
5000	193.50 ± 21.50
10,000	313.00 ± 14.00
15,000	465.00 ± 45.00
25,000	1,190.00 ± 90.00

The results indicated that for oil in brine concentrations of 1000, 5000, 10,000, 15,000, and 25,000 ppm, only the 25,000 ppm concentration yielded a TOG value sufficiently close to the target (1190 ± 90.00 ppm), approaching the expected value of 1000 ppm. These data demonstrate that, due to the high viscosity of the oil, there is significant difficulty in achieving homogenization and complete incorporation of the oil into the brine, resulting in notable losses throughout the process. This limitation is particularly evident during agitation with the Polytron, due to the oil’s strong adhesion to the equipment’s shaft and the walls of the beaker used for preparation. Furthermore, after transferring the oily water to the separatory funnel and allowing it to stand for one hour, a considerable amount of oil remains adhered to the funnel’s inner walls, contributing to additional losses.

Based on the theoretical concentration of 25,000 ppm of petroleum, resulting in actual concentrations of ∼1200 ppm and ∼4,500 ppm, with and without SPAM. The increase in oil concentration in the presence of SPAM can be associated with the chemical interaction between the ions dissolved in the saline water and the sulfonate groups (SO_3_^–^) present in the SPAM structure, which are highly hydrophilic and remain dissociated in solution, especially in saline media. In water with a high concentration of dissolved ions (such as Na^+^, Ca^2+^, Mg^2+^), these sulfonate groups can interact strongly with the cations present, promoting electrostatic shielding effects or even the formation of complexes that modify the conformation of the polymer chain. These interactions can contribute to the incorporation of oil into the brine, leading to a higher concentration of oil in the prepared water.^39,44^

These results highlighted the impact of SPAM on oil retention in the O/W emulsion, demonstrating the need to investigate its influence on the performance of flocculants in oily water treatment, as well as to investigate the influence of the demulsifier added individually and in the presence of SPAM. With these studies we sought to understand the interactions involved, providing a clearer view of the conditions that favor separation and improve the quality of oily water after treatment.

### 3.3 Evaluation of the Demulsifier Individually, with and without SPAM by the Jar Test

Flocculation tests were initiated with demulsifiers to verify if the isolated use of these products could interfere with TOG values. Tables 3 and 4 present the TOG removal efficiency using demulsifier, respectively without and with the addition of SPAM. The efficiencies were ranked in comparison to the blank test, which was always performed with the first and last oily water fractions removed from the separatory funnel, to verify good dispersion between the components. As expected, TOG values were not significantly influenced by the presence of the demulsifier, without SPAM (Table 3), at the three concentrations tested. The sample without demulsifier showed an average efficiency of 51.12%, while the concentrations of 50, 175, and 350 ppm resulted in efficiencies of 41.76%, 43.58%, and 49.43%, respectively, with all values falling within the measurement error. The same behavior was observed in Table 4, with SPAM, where the average efficiency values also remained within the measurement error. The ineffectiveness of the demulsifier in separating the O/W emulsion can be better understood from the concept of hydrophilic–lipophilic balance (HLB), which describes the relationship between the hydrophilic (affinity for water) and lipophilic (affinity for oil) portions of a surfactant. In the case of the demulsifier used, its greater interaction with the oil phase compared to the aqueous phase suggests that the HLB of the product was low, making it difficult to break the O/W emulsion, since the demulsifier cannot interact effectively with water to promote separation. In O/W emulsions, for effective separation the surfactant must be able to weaken the interactions between the oil particles and the aqueous phase. Conversely, a surfactant with a higher HLB would be more hydrophilic, able to interact more easily with the aqueous phase, promoting better separation.^45,46^ The efficiency of the demulsifier depends on its ability to promote competitive displacement at the interface; therefore, when its interaction with the oil phase is excessive, this mechanism of action becomes limited.^47^

**Table 3:** TOG Removal Efficiency Using a Demulsifier without SPAM

Bottle	Demulsifier (ppm)	Before Jar test (ppm)	After Jar test (ppm)	Average efficiency (%)
1	0	1095.00 ± 5.00	535.00 ± 55.00	51.12 ± 5.25
2	50	1175.00 ± 25.00	685.00 ± 45.00	41.76 ± 2.59
3	175	1150.00 ± 30.00	650.00 ± 60.00	43.58 ± 3.75
4	350	1265.00 ± 45.00	640.00 ± 30.00	49.43 ± 0.57
5	0	1235.00 ± 55.00	630.00 ± 30.00	48.78 ± 4.71

**Table 4:** TOG Removal Efficiency Using Demulsifier with SPAM

Bottle	Demulsifier (ppm)	Before Jar test (ppm)	After Jar test (ppm)	Average efficiency (%)
1	0	4260.00 ± 50.00	2435.00 ± 145.00	42.79 ± 4.08
2	50	4795.00 ± 35.00	2405.00 ± 175.00	49.81 ± 4.02
3	175	4335.00 ± 85.00	2385.00 ± 15.00	44.97 ± 0.73
4	350	4770.00 ± 20.00	2355.00 ± 55.00	50.63 ± 0.95
5	0	4215.00 ± 105.00	2425.00 ± 65.00	42.39 ± 2.98

### 3.4 TOG Reduction Efficiency of Flocculants A and B with and without 450 ppm of SPAM, without Demulsifier

Figure 1 shows the results of a jar test of flocculant A with and without SPAM, without demulsifier. The efficiency of the process increased progressively with rising flocculant concentration. Without the addition of SPAM (black bar), at 50 ppm of flocculant, no efficiency was observed when comparing the result with oily water without flocculant (0 ppm), indicating an insufficient dosage to agglomerate the oil particles dispersed in the oily water. Between 100 and 500 ppm, the efficiency of flocculant A increased progressively, from 75% at 100 ppm to 91% at 500 ppm, demonstrating that the increase in concentration favored greater agglomeration of the dispersed oil particles.

**Figure 1:**
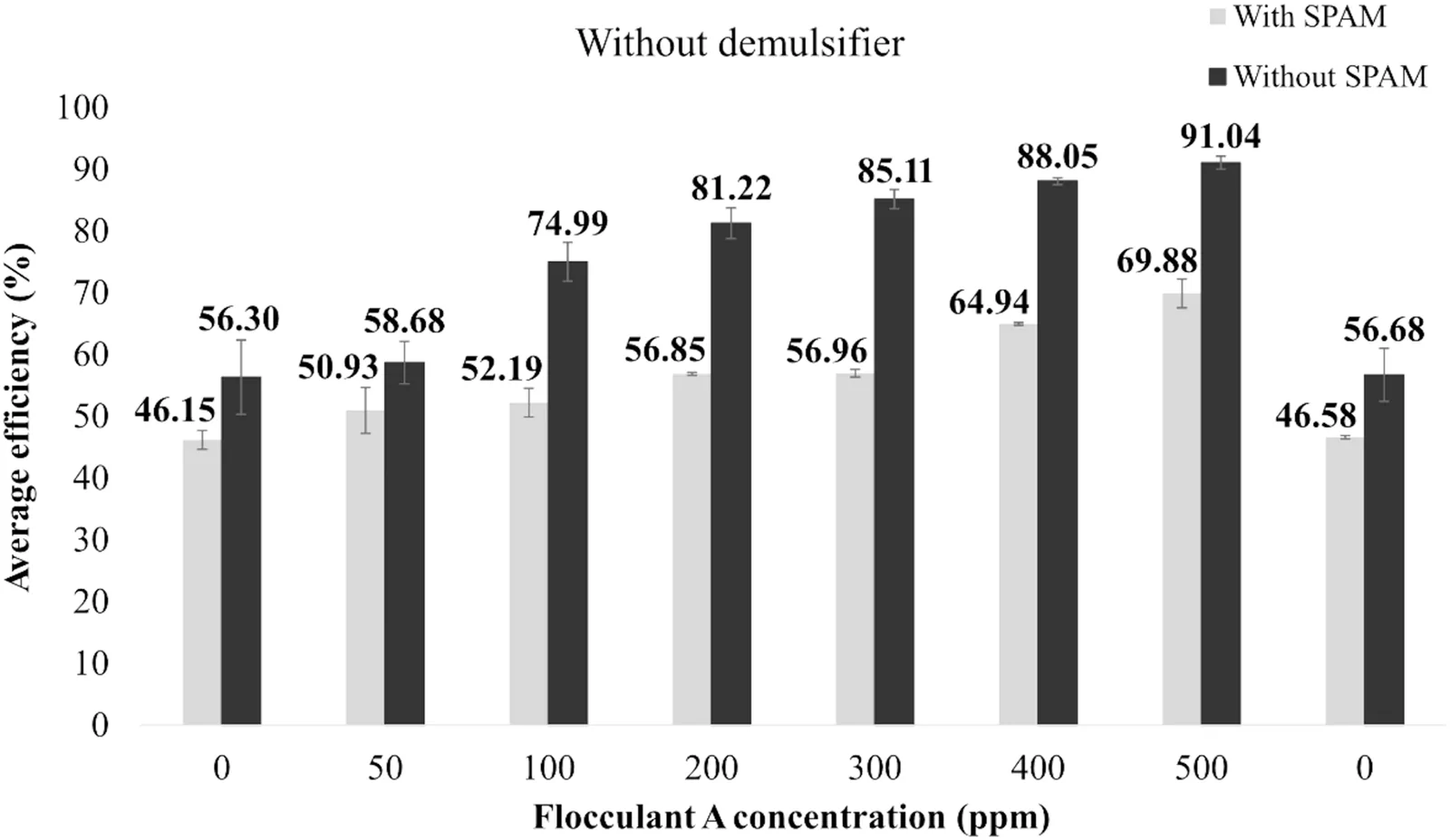
TOG reduction efficiency for flocculant A with and without 450 ppm of SPAM, without demulsifier.

In the presence of SPAM (gray bar), the same trend of increased efficiency with increasing flocculant concentration was observed. However, the increase in efficiency was more limited. At the lowest concentrations (50 and 100 ppm,), efficiencies of 51% and 52% were obtained, respectively, a result very close to those of the system without flocculant (46%). At intermediate concentrations of 200 and 300 ppm of flocculant A, the increase in efficiency was not very substantial, reaching ∼57% in both cases. At higher concentrations (400 and 500 ppm) there was an increase in efficiency (65 and 70%, respectively). However, these values were still much lower than those achieved without the presence of SPAM.

One of the mechanisms proposed for the stabilization of oil droplets by sulfonated polyacrylamide is the increase in the viscosity of the continuous phase, which reduces the frequency of collisions between droplets.^48^

Another mechanism would be the formation of steric layers around the droplets, preventing their approach and coalescence. Under these conditions, the polymer tends to adsorb around the droplets, forming a protective layer that hinders coalescence. As a result, the flocculant’s performance at the oil–water interface was severely impaired, even when used at relatively high concentrations.^39,44,49^

Figure 2 shows the results of the jar test for flocculant B with and without SPAM, without demulsifier. The same trend shown by flocculant A was observed for flocculant B, namely an increase in efficiency with rising concentration. However, this increase was more pronounced, with efficiency of 84% at a concentration of 50 ppm. At 100 ppm of flocculant B, the same performance (92%) as flocculant A at 500 ppm (91%) was achieved. Using only 200 ppm of flocculant B, efficiency very close to the maximum (98%) was achieved.

**Figure 2:**
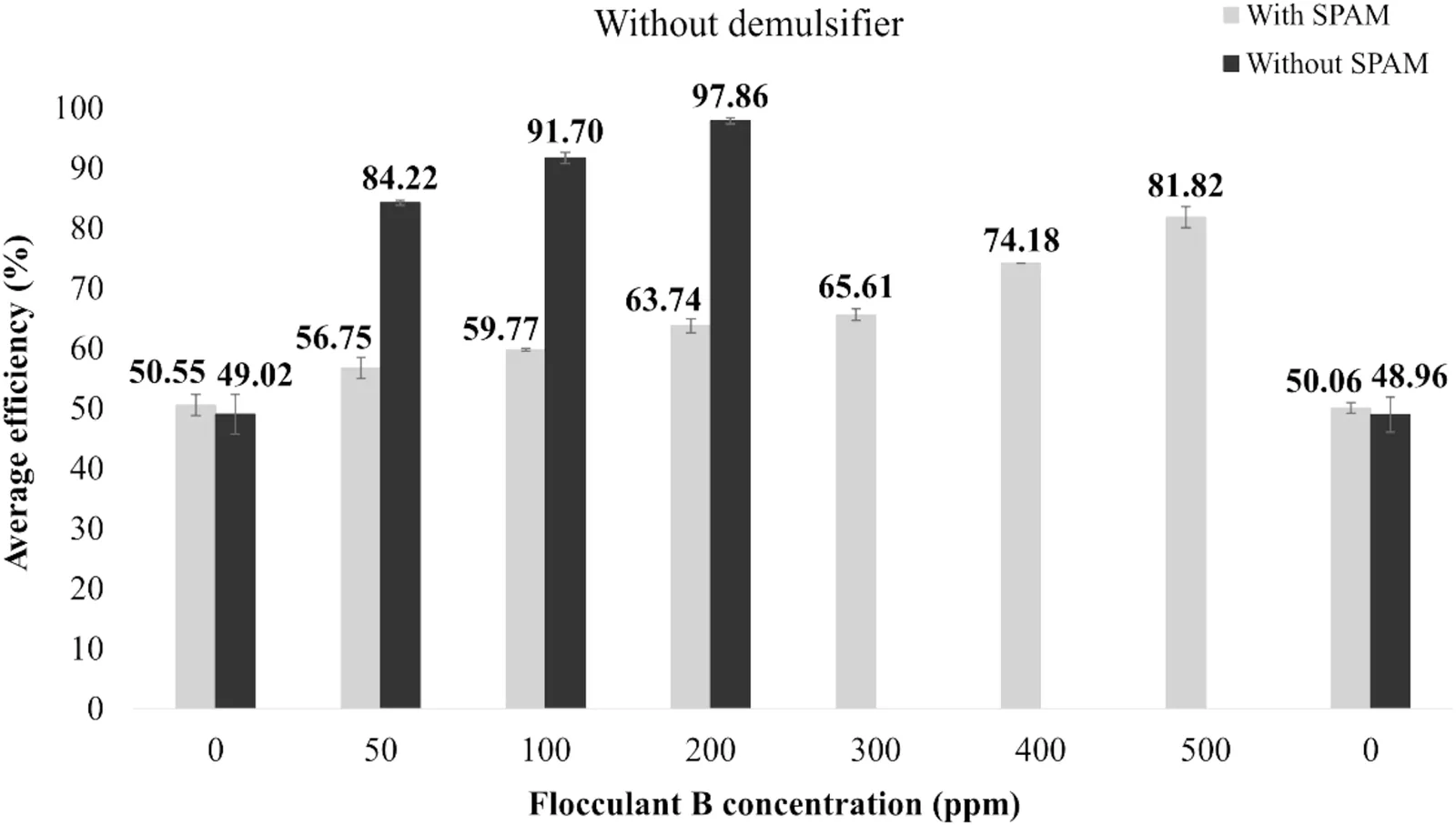
TOG reduction efficiency for flocculant B with and without 450 ppm of SPAM, without demulsifier.

In the presence of SPAM, behavior similar to that observed with flocculant A was noted; that is, a loss of efficiency occurred due to the presence of SPAM. For example, at 200 ppm of flocculant B, the efficiency dropped from 98% to 64%, respectively, without and with 450 ppm of SPAM. However, the efficiencies remained higher than those observed for flocculant A at all concentrations.

The flocculation capacity of flocculants A and B may be related to the relatively low mass charge density of these molecules (flocculant A = 0.39 mequiv/g and flocculant B = 0.22 mequiv/g). According to the literature,^50^ polymers with low charge density tend to be more effective than nonionic polymers because the residual charges in their chains promote a slight electrostatic repulsion in the medium. The repulsion promoted by the low charge density contributes to the elongation of the polymer chains, increasing their flexibility and favoring the formation of bridges between particles. This extended conformation reduces the chance that multiple chain segments will adsorb onto a single particle, allowing the free chain segments to bind to other neighboring particles.^50^ Therefore, the low charge density values observed for flocculants A and B can be interpreted not as a limitation, but as a characteristic that favors the flocculation mechanism through polymer bridges. This explains why, even with low charge density, these flocculants showed satisfactory performance in the aggregation of oily particles in solution, especially flocculant B, which had a slightly lower charge density than flocculant A and was more efficient.

Increasing the concentration of flocculants favored polymer chain bridging, meaning greater partial adsorption of long polymer chains on the surface of the dispersed droplet, promoting better flocculant efficiency. However, for polymer bridging to be effective, the polymer concentration must be carefully controlled, as very low concentrations result in an insufficient number of interactions, while excessive concentrations can lead to surface saturation, causing restabilization of the colloidal system and impairing aggregation efficiency.^51^

### 3.5 TOG Reduction Efficiency of Flocculants A and B, Varying the Demulsifier Concentration, with and without 450 ppm of SPAM

The presence of production chemicals can influence the performance of flocculants. Since demulsifiers are added to petroleum in a step prior to water treatment, these additives are present in the system during the flocculation process. Therefore, to better understand the effect of the demulsifier on the performance of flocculants A and B, we conducted experimental tests with the combined application of these two types of products, varying the concentration of the demulsifier.

Figures 3 and 4 show the influence of the demulsifier on the efficiency of flocculants A and B, respectively, with and without 450 ppm of SPAM. For flocculant A (Figure 3), in tests with and without demulsifier (black bar), efficiency gradually increased with increasing concentrations of the systems in which lower concentrations of demulsifier were added. However, this increase in efficiency gradually declined with increasing demulsifier concentration. Between flocculant concentrations of 50 and 200 ppm, these increases ranged from 59 to 81%, 55 to 71%, and 52 to 65%, respectively, for systems with 0, 50, and 175 ppm of demulsifier. As previously discussed, the demulsifier exhibited better interaction with the oil, defined by the HLB concept and the charge density results, where the demulsifier showed a charge density quite similar to that of the oily water. In this case, the demulsifier may have prevented the adsorption of the flocculant at the oil–water interface, reducing its performance.

**Figure 3:**
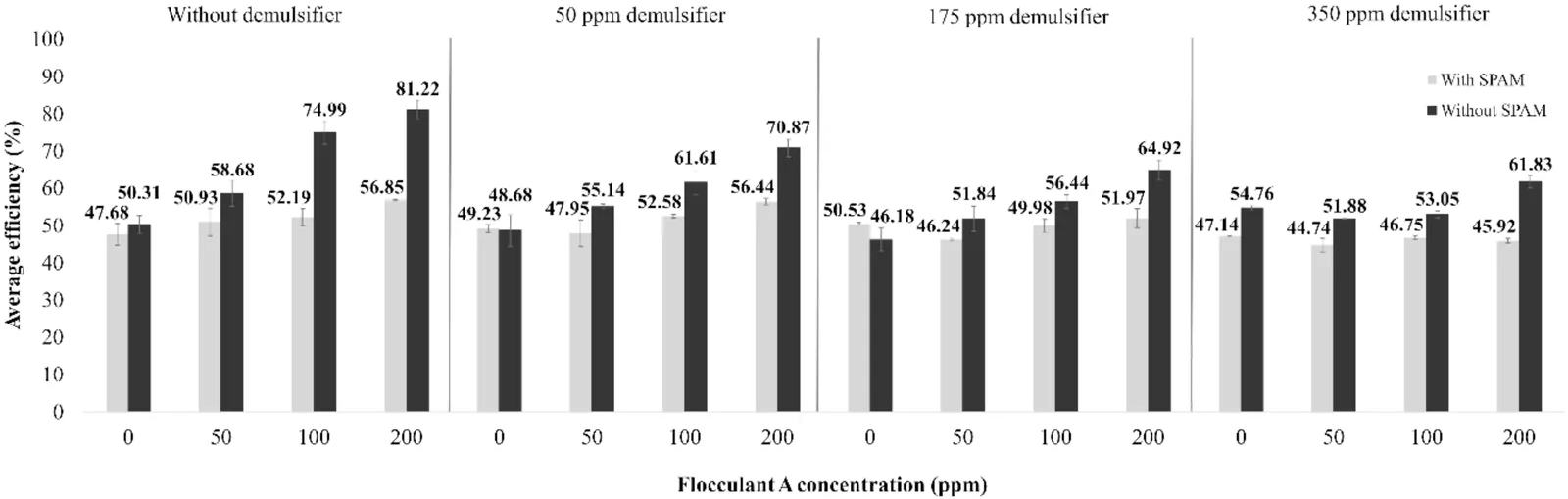
TOG reduction efficiency for flocculant A at concentrations of 0, 50, 100, and 200 ppm, with different demulsifier concentrations (0, 50, 175, and 350 ppm) without and with 450 ppm of SPAM.

**Figure 4:**
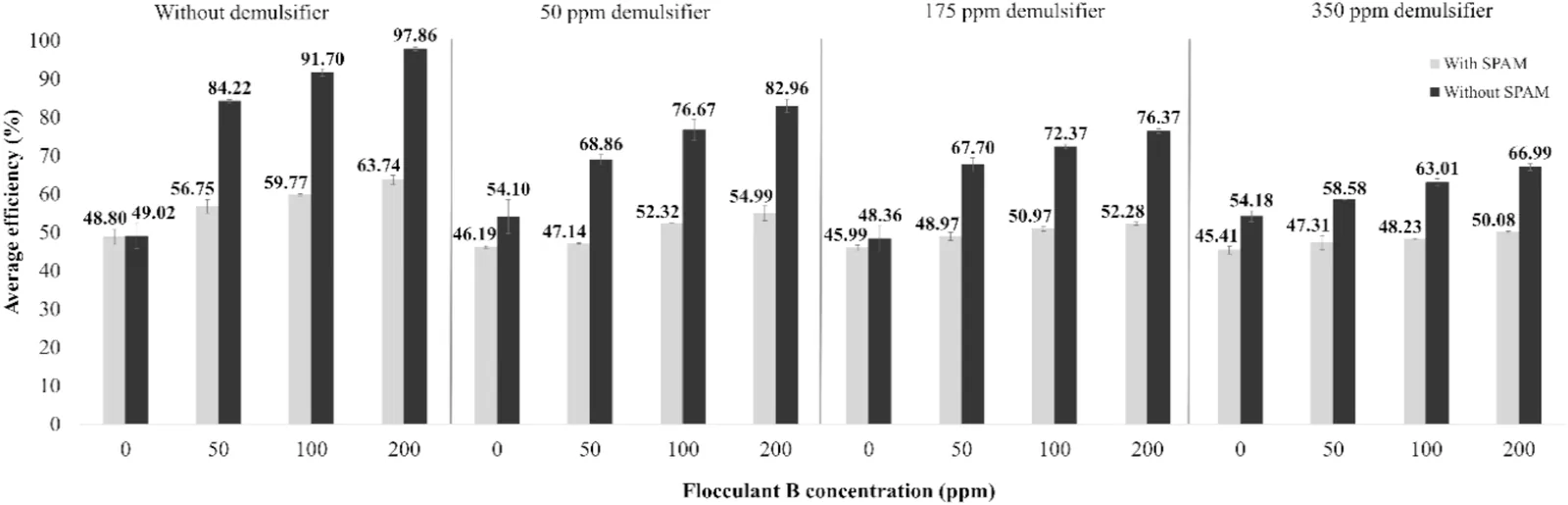
TOG reduction efficiency for flocculant B at concentrations of 0, 50, 100, and 200 ppm, with different demulsifier concentrations (0, 50, 175, and 350 ppm) without and with 450 ppm of SPAM.

With 350 ppm of demulsifier, the impact on flocculant efficiency was even more pronounced. The flocculant lost efficiency at virtually all concentrations evaluated (50, 100, and 200 ppm). This trend indicates that the demulsifier’s interference becomes more pronounced as its concentration increases, compromising the flocculant’s action and hindering the removal of oil from oily water. Under these conditions, only the highest flocculant concentration (200 ppm) showed some efficiency, reaching 62%. At flocculant A concentrations of 50 and 100 ppm, the presence of 350 ppm of demulsifier rendered the process completely inefficient.

Flocculant B (Figure 4), in tests with demulsifier and without SPAM (black bar), followed the same trend of performance loss with increasing concentration of demulsifier A. In the comparison of flocculant B concentrations of 50 and 200 ppm, the efficiency increases were 84 to 98%, 67 to 83%, 68 to 76%, and 58 to 67%, respectively, for systems with 0, 50, 175, and 350 ppm of demulsifier. However, the performance of flocculant B was superior to that of flocculant A at all demulsifier concentrations: comparing concentrations of 200 ppm of flocculants A and B, efficiencies of 81 and 98%, 71 and 83%, 65 and 76%, and 62 and 67% were observed, respectively, for the addition of 0, 50, 175, and 350 ppm of demulsifier.

When evaluating the interaction of SPAM together with the demulsifier on the performance of flocculants A and B in the treatment of oily water, we observed that for flocculant A (Figure 3—gray bar) performance was suppressed in the presence of SPAM and the demulsifier at the concentrations evaluated. That is, the combined effect of the demulsifier with SPAM significantly reduced the performance of the flocculants, due to the electrostatic shielding generated by SPAM in the brine and the presence of the demulsifier at the oil–water interface. This effect became more pronounced with increasing demulsifier concentration, at which point flocculant A was completely ineffective, even at a concentration of 200 ppm.

As for flocculant B (Figure 4—gray bar), which was able to maintain some efficiency with the demulsifier and SPAM added individually, the action was almost completely suppressed in the presence of these two additives together.

### 3.6 TOG Reduction Efficiency of Flocculants A and B with 50 ppm of Demulsifier, without and with 450 ppm of SPAM

Since the influence of the demulsifier on the efficiency of the flocculants was observed, we performed analyses using the lowest concentration of demulsifier (50 ppm) and increasing the concentration of flocculants to 300, 400, and 500 ppm, with the aim of verifying whether the flocculants improved performance in the presence of the demulsifier when used at higher concentrations.

Figure 5 shows the TOG reduction efficiency of flocculant A. Increasing the flocculant concentration to 500 ppm led to an increase in efficiency, reaching 83% without the addition of SPAM (black bar). However, this value was lower than that obtained in the absence of demulsifier, when the efficiency reached 91% with the same flocculant concentration (Figure 1). This behavior indicated that, even at low concentrations, the demulsifier at the interface limits the adsorption of polymer chains onto the oil droplets, hindering the formation of bridges between the particles.

**Figure 5:**
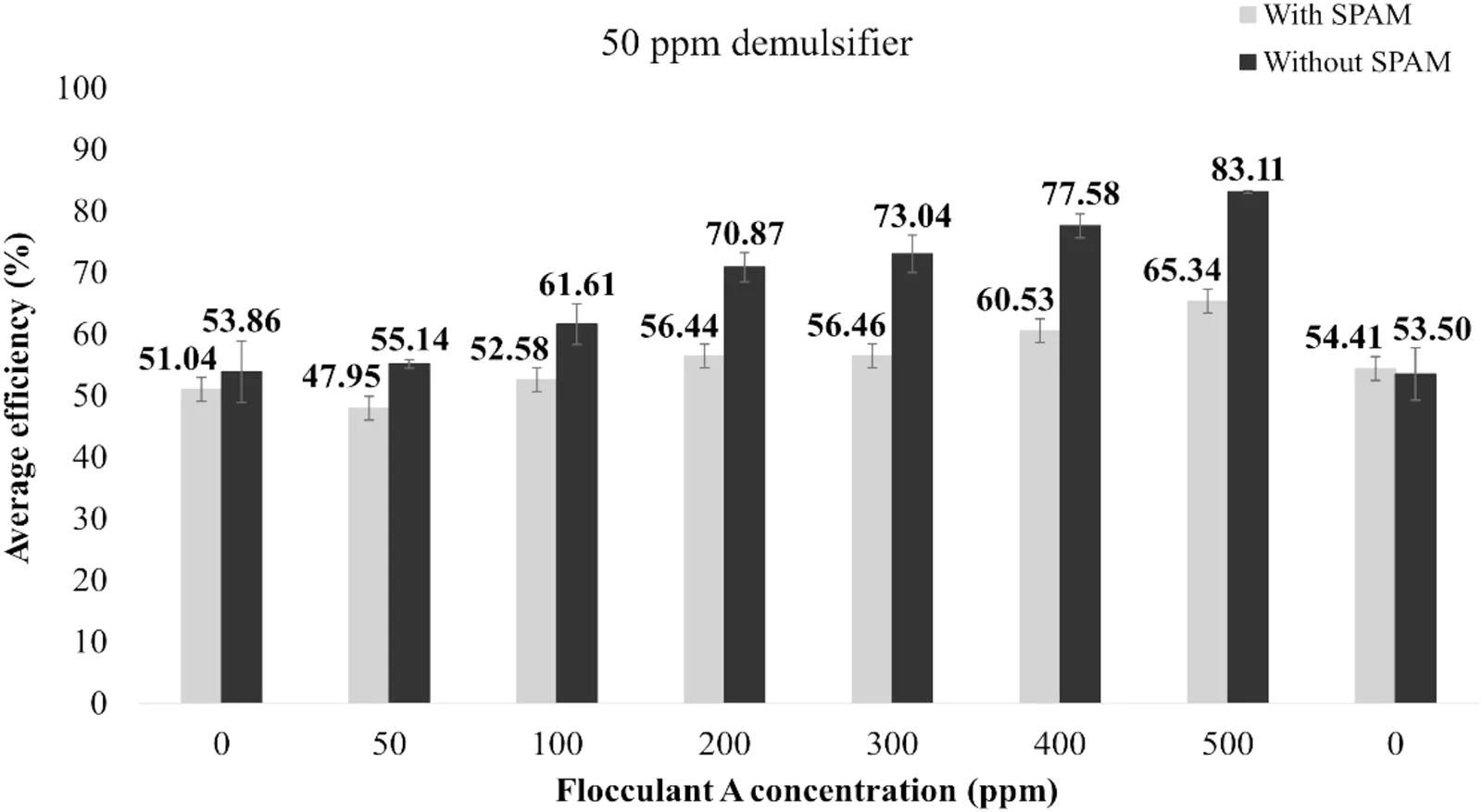
TOG reduction efficiency for flocculant A at concentrations of 0 to 500 ppm, with the addition of 50 ppm of demulsifier, without and with 450 ppm of SPAM.

For flocculant B without the addition of SPAM (black bar) (Figure 6), better efficiencies were observed in comparison with flocculant A, which had already been observed previously. However, due to the influence of the demulsifier, maximum efficiency was not achieved even at 500 ppm (91%), whereas without demulsifier, flocculant B had achieved 98% efficiency (Figure 2) with only 200 ppm. At higher concentrations, the action of the flocculant at the interface overrides the action of the demulsifier, achieving higher efficiency values.

**Figure 6:**
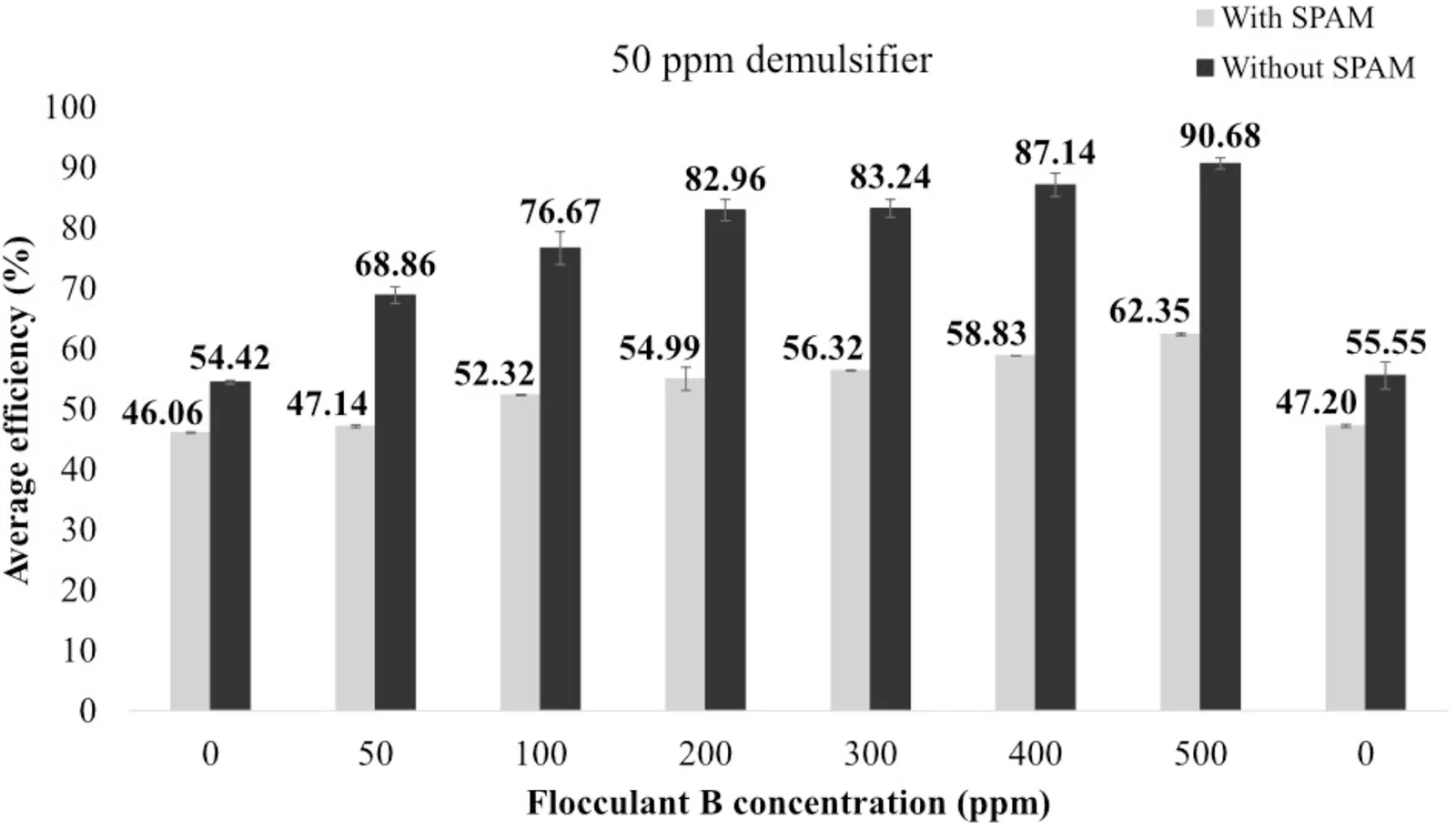
TOG reduction efficiency for flocculant B at concentrations of 0 to 500 ppm, with the addition of 50 ppm of demulsifier, without and with 450 ppm of SPAM.

With the presence of SPAM (gray bar), we observed for flocculant A (Figure 5) that there was no increase in efficiency at concentrations up to 300 ppm. There was slight and gradual improvement with increasing flocculant concentrations to 400 and 500 ppm. However, even at a concentration of 500 ppm, the efficiency was only 65%. In this case, flocculant A showed similar efficiency to the test without demulsifier and with SPAM (70%) at the same concentration tested (Figure 1), indicating that the influence of SPAM on the flocculant’s performance was more pronounced than that of the demulsifier at the concentrations used. For flocculant B (Figure 6), similar behavior was observed, that is, in the presence of 50 ppm of demulsifier and 450 ppm of SPAM (gray bar), flocculant B lost its competitiveness in relation to flocculant A, as both became practically ineffective at the concentrations studied.

The results show the type and concentration of flocculant needed to achieve high levels of water treatment as a function of the amount of demulsifier present in the oil. In this case, very high concentrations of flocculant B should be used, in the presence of the demulsifier, to obtain better flocculant performance.

### 3.7 Evaluation of the Efficiency of Flocculants A and B in Oily Water, with and without 450 ppm of SPAM, at the Same Oil Concentration in the Water

First, we adjusted the oily water preparation method to obtain an oil concentration of approximately 1200 ppm in the brine, while maintaining a SPAM concentration of 450 ppm. The amount of petroleum added to the brine had to be gradually reduced from 25,000 to 5000 ppm, which achieved an oil concentration close to 1200 ppm. This made it possible to compare systems with and without SPAM at the same petroleum concentration in the brine.

Table 5 shows the efficiency results of flocculant A in the presence of SPAM, demonstrating that efficiency was greater in samples with lower oil concentration in the water (∼1200 ppm). Without demulsifier, 85% efficiency was obtained using 500 ppm of flocculant, while with oily water of ∼4500 ppm the efficiency obtained was 70%. This trend of higher efficiency of flocculant A with lower oil content in the water was consistent across all demulsifier concentrations tested (0, 50, 175, and 350 ppm). However, even with the improvement associated with the lower oil concentration, when comparing the tests with and without SPAM in oily water with ∼1200 ppm of oil, we observed that the samples without SPAM and without demulsifier achieved higher efficiencies (91%) with 500 ppm of flocculant. The same trend was observed in the presence of 50 ppm of demulsifier. However, with the increases in demulsifier concentration to 175 and 350 ppm, the efficiency values became similar to those of SPAM, regardless of the oil content in the synthetic oily water, which was observed for all flocculant concentrations.

**Table 5:** Oil Removal Efficiency Using Flocculant A in Three Types of Synthetic Oily Water: without SPAM ∼1200 ppm Oil, with 450 ppm of SPAM at ∼1200 ppm Oil, and with 450 ppm of SPAM at ∼4500 ppm Oil

		Oily Water		
		Without SPAM	With SPAM	With SPAM
		(∼1200 ppm)	(∼1200 ppm)	(∼4500 ppm)
Demulsifier (ppm)	Flocculant A (ppm)	Average efficiency (%)		
0	0	56.30 ± 6.00	49.16 ± 0.80	46.15 ± 1.53
	50	58.68 ± 3.43	54.18 ± 0.74	50.93 ± 3.72
	100	74.99 ± 3.16	63.43 ± 2.49	52.19 ± 2.33
	200	81.22 ± 2.48	69.41 ± 1.91	56.85 ± 0.26
	300	85.11 ± 1.55	75.88 ± 0.50	56.96 ± 0.62
	400	88.05 ± 0.55	79.12 ± 3.32	64.94 ± 0.23
	500	91.04 ± 1.04	85.22 ± 0.72	69.88 ± 2.33
	0	56.68 ± 4.29	48.26 ± 0.21	46.58 ± 0.26
50	0	53.86 ± 4.99	48.50 ± 0.23	51.04 ± 1.80
	50	55.14 ± 0.69	50.58 ± 1.39	47.95 ± 3.54
	100	61.61 ± 3.28	56.03 ± 2.70	52.58 ± 0.48
	200	70.87 ± 2.37	61.17 ± 0.37	56.44 ± 0.82
	300	73.04 ± 3.04	68.13 ± 1.47	56.46 ± 0.11
	400	77.58 ± 1.95	73.31 ± 2.47	60.53 ± 1.47
	500	83.11 ± 0.23	77.71 ± 1.49	65.34 ± 0.73
	0	53.50 ± 4.25	47.83 ± 0.25	54.41 ± 1.61
175	0	46.18 ± 3.10	48.08 ± 3.54	50.53 ± 0.40
	50	51.84 ± 3.40	49.20 ± 1.20	46.24 ± 0.44
	100	56.44 ± 1.83	52.58 ± 0.64	49.98 ± 1.78
	200	64.92 ± 2.71	56.73 ± 1.61	51.97 ± 2.58
	0	50.59 ± 3.41	51.16 ± 1.56	49.88 ± 2.58
350	0	54.76 ± 0.62	47.08 ± 2.92	47.14 ± 0.10
	50	51.88 ± 0.12	45.60 ± 3.22	44.74 ± 1.80
	100	53.05 ± 0.98	46.19 ± 0.73	46.75 ± 0.46
	200	61.83 ± 1.69	47.74 ± 1.08	45.92 ± 0.64
	0	51.91 ± 3.41	47.57 ± 1.23	48.97 ± 0.76

Table 6 presents the average oil removal efficiency results for flocculant B in the presence of SPAM, confirming the same trend observed for flocculant A. Without demulsifier, 92% efficiency was obtained using 500 ppm of flocculant, while with oily water of ∼4500 ppm the efficiency obtained was 82%. However, even with the improvement associated with the lower oil concentration, when comparing the tests with and without SPAM in oily water with ∼1200 ppm of oil, we observed that the samples without SPAM and without demulsifier achieved higher efficiencies (98%) already with 200 ppm of flocculant. The high efficiency of flocculant B can still be observed in the presence of 50 ppm of demulsifier. However, when the demulsifier concentration is increased to 175 and 350 ppm, the efficiency values become similar for both oily water concentrations. The only exception occurred with 200 ppm of flocculant and 175 ppm of demulsifier, under which the efficiency was 62% at ∼1200 ppm and 52% at ∼4500 ppm. These results validated the discussion previously conducted for both flocculant A and B, when synthetic waters with different oil contents were used for samples with and without SPAM.

**Table 6:** Oil Removal Efficiency Using Flocculant B in Three Types of Oily Water: without SPAM ∼1200 ppm of Oil, with 450 ppm of SPAM at ∼1200 ppm of Oil, and with 450 ppm of SPAM at ∼4500 ppm of Oil

		Oily Water		
		Without SPAM	With SPAM	With SPAM
		(∼1200 ppm)	(∼1200 ppm)	(∼4500 ppm)
Demulsifier (ppm)	Flocculant B (ppm)	Average efficiency (%)		
0	0	49.02 ± 3.29	43.59 ± 0.74	50.55 ± 1.75
	50	84.22 ± 0.40	61.43 ± 1.43	56.75 ± 1.75
	100	91.70 ± 0.93	71.03 ± 1.49	59.77 ± 0.23
	200	97.86 ± 0.50	79.33 ± 0.97	63.74 ± 1.17
	300	-	83.31 ± 1.09	65.61 ± 0.96
	400	-	86.94 ± 0.87	74.18 ± 0.02
	500	-	91.93 ± 0.86	81.82 ± 1.76
	0	48.96 ± 2.92	43.82 ± 0.44	50.06 ± 0.88
50	0	54.42 ± 0.32	46.28 ± 0.56	46.06 ± 0.12
	50	68.86 ± 1.38	53.28 ± 0.57	47.14 ± 0.23
	100	76.67 ± 2.72	60.29 ± 0.61	52.32 ± 0.03
	200	82.96 ± 1.77	67.36 ± 0.14	54.99 ± 1.92
	300	83.24 ± 1.49	73.84 ± 0.97	56.32 ± 0.09
	400	87.14 ± 1.92	78.30 ± 0.61	58.83 ± 0.01
	500	90.68 ± 0.92	81.98 ± 1.22	62.35 ± 0.19
	0	55.55 ± 2.25	46.03 ± 0.45	47.20 ± 0.24
175	0	48.36 ± 3.36	46.14 ± 1.69	45.99 ± 0.68
	50	67.70 ± 1.76	50.39 ± 0.39	48.97 ± 1.03
	100	72.37 ± 0.60	55.94 ± 0.21	50.97 ± 0.64
	200	76.37 ± 0.61	61.81 ± 1.31	52.28 ± 0.38
	0	55.52 ± 0.45	46.38 ± 1.06	46.99 ± 1.54
350	0	54.18 ± 1.46	45.96 ± 0.65	45.41 ± 1.04
	50	58.58 ± 0.01	46.67 ± 0.16	47.31 ± 1.91
	100	63.01 ± 0.96	47.36 ± 1.02	48.31 ± 0.15
	200	66.99 ± 0.84	50.61 ± 0.20	50.08 ± 0.25
	0	50.81 ± 3.48	46.36 ± 0.56	48.69 ± 1.31

### 3.8 Determination of the Size and Particle Size Distribution of Oily Water by Mastersizer

Particle size analysis was used as a complementary measure to the oil removal efficiency tests, using oily water with ∼1200 ppm, in the absence and presence of SPAM, allowing a more detailed evaluation of the performance of the tested flocculants, especially regarding floc formation. Among the parameters evaluated, D10 and D50 showed inconsistent variations, probably associated with the high viscosity of the heavy oil used. Elevated viscosity hinders proper particle dispersion, preventing the laser from adequately penetrating the suspension and accurately interpreting the scattering pattern. As a result, measurements for small (D10) and medium particles (D50) become imprecise, whereas larger particles (D90) still generate a more reliable signal.^52^ Thus, D90 was considered the most representative parameter for comparison between the flocculants.

It was also observed that, despite the high viscosity of the oil, particle size analysis demonstrates the good reproducibility of the oily water preparation method, given the similar values obtained for samples prepared in separate batches.

Table 7 presents the particle size (D90) values obtained under different experimental conditions, without SPAM and with variations in demulsifier concentrations (0 to 350 ppm) for flocculants A and B at concentrations from 0 to 500 ppm. For flocculant A, the results showed a clear correlation between particle size (D90) and average oil removal efficiency. Increasing the concentration of flocculant A favored both average efficiency and the formation of larger aggregates, as observed at 500 ppm, where D90 reached 926.5 μm and removal efficiency was 91%. On the other hand, the addition of the demulsifier compromised these parameters, resulting in a decrease in average efficiency and a reduction in particle size. This effect was most evident in the combination of 350 ppm of demulsifier with 50 ppm of flocculant, where the efficiency dropped to 52% and the D90 declined significantly to 76 μm, indicating a limitation in the formation of aggregates large enough for efficient sedimentation.

**Table 7:** Particle Size (D90) and Average Oil Removal Efficiency without SPAM, with Different Concentrations of Demulsifier and Flocculant A and B

		Flocculant A		Flocculant B	
Demulsifier concentration (ppm)	Flocculant concentration (ppm)	Average efficiency (%)	Particle Size (D90) (μm)	Average efficiency (%)	Particle Size (D90) (μm)
0	0	52.39 ± 0.24	86.9 ± 1.8	49.02 ± 3.29	49.6 ± 1.5
	50	58.68 ± 3.43	154.5 ± 4.5	84.22 ± 0.40	705.0 ± 10.0
	100	74.99 ± 3.16	358.5 ± 13.5	91.70 ± 0.93	966.0 ± 12.0
	200	81.22 ± 2.48	539.5 ± 13.5	97.86 ± 0.50	1385.0 ± 15.0
	300	85.11 ± 1.55	723.0 ± 14.0	-	-
	400	88.05 ± 0.55	808.5 ± 12.5	-	-
	500	91.04 ± 1.04	926.5 ± 47.5	-	-
50	0	53.86 ± 4.99	84.5 ± 3.1	54.42 ± 0.32	93.6 ± 0.2
	50	55.14 ± 0.69	116.5 ± 5.5	68.86 ± 1.38	266.0 ± 1.0
	100	61.61 ± 3.28	189.0 ± 5.0	76.67 ± 2.72	430.0 ± 17.0
	200	70.87 ± 2.37	280.0 ± 18.0	82.96 ± 1.77	619.0 ± 13.0
	300	73.04 ± 3.04	337.0 ± 10.0	83.24 ± 1.49	641.5 ± 0.5
	400	77.58 ± 1.95	449.5 ± 37.5	87.14 ± 1.92	747.5 ± 10.5
	500	83.11 ± 0.23	638.0 ± 13.0	90.68 ± 0.92	899.0 ± 18.0
175	0	50.59 ± 3.41	68.2 ± 2.1	48.36 ± 3.36	53.3 ± 1.35
	50	51.84 ± 3.40	79.7 ± 2.95	67.70 ± 1.76	266.0 ± 4.0
	100	56.44 ± 1.83	122.0 ± 5.0	72.37 ± 0.60	330.0 ± 8.0
	200	64.92 ± 2.71	216.0 ± 4,0	76.37 ± 0.61	430.5 ± 3.5
350	0	51.91 ± 1.52	79.6 ± 8.6	54.18 ± 1.46	87.1 ± 2.45
	50	51.88 ± 0.12	75.9 ± 5.65	58.58 ± 0.01	159.5 ± 2.5
	100	53.05 ± 0.98	91.8 ± 1.9	63.01 ± 0.96	201.5 ± 8.5
	200	61.83 ± 1.69	190.0 ± 3.0	66.99 ± 0.84	242.0 ± 1.0

The results for flocculant B followed the same trend observed for flocculant A, where increasing the concentration favored both the average oil removal efficiency and the formation of larger aggregates. However, the values obtained for flocculant B were superior, standing out with 200 ppm of the flocculant without demulsifier, where the D90 reached 1385.0 μm and the efficiency reached 98%. On the other hand, the addition of the demulsifier also negatively impacted these parameters, reducing both efficiency and particle size; the combination of 350 ppm of demulsifier with 50 ppm of flocculant achieved efficiency of 58.58% and a reduced D90 of 159.5 μm.

The particle size (D90) results in the presence of 450 ppm of SPAM for both flocculants A and B are presented in Table 8. For flocculant A, in the presence of SPAM, the particle size was considerably smaller compared to the tests without SPAM (Table 7). Although increasing the concentration of flocculant A promoted a progressive increase in both removal efficiency and particle size (D90), the maximum value obtained was lower than that observed in the tests without SPAM. In the test with 500 ppm of flocculant and without demulsifier, the average efficiency reached 85% with a D90 of 739.0 μm. The presence of the demulsifier, in turn, exerted a negative effect similar to that observed in the tests without SPAM, gradually reducing both the efficiency and the size of the aggregates formed. This impact was even more pronounced due to the combined action with SPAM, which potentiated this adverse effect, hindering the formation of larger flocs. This behavior was most evident in the combination of 350 ppm of demulsifier with 50 ppm of flocculant, where the efficiency decreased to 45% and the D90 was 43.20 μm.

**Table 8:** Particle Size (D90) and Average Oil Removal Efficiency with 450 ppm of SPAM, with Different Concentrations of Demulsifier and Flocculant A and B

		Flocculant A		Flocculant B	
Demulsifier concentration (ppm)	Flocculant concentration (ppm)	Average efficiency (%)	Particle Size (D90) (μm)	Average efficiency (%)	Particle Size (D90) (μm)
0	0	49.16 ± 0.80	50.3 ± 4.95	43.59 ± 0.74	41.6 ± 1.70
	50	54.18 ± 0.74	95.3 ± 1.65	61.43 ± 1.43	185.5 ± 3.50
	100	63.43 ± 2.49	205.0 ± 5.0	71.03 ± 1.49	290.5 ± 3.50
	200	69.41 ± 1.91	266.5 ± 2.5	79.33 ± 0.97	498.5 ± 5.50
	300	75.88 ± 0.50	423.5 ± 4.5	83.31 ± 1.09	656.0 ± 7.00
	400	79.12 ± 3.32	490.0 ± 6.0	86.94 ± 0.87	751.0 ± 13.00
	500	85.22 ± 0.72	739.0 ± 14.0	91.93 ± 0.86	933.5 ± 9.50
50	0	48.50 ± 0.23	56.4 ± 2.65	46.28 ± 0.56	44.1 ± 0.50
	50	50.58 ± 1.39	74.5 ± 4.45	53.28 ± 0.57	95.2 ± 2.30
	100	56.03 ± 2.70	126.0 ± 5.0	60.29 ± 0.61	167.5 ± 1.50
	200	61.17 ± 0.37	184.5 ± 1.5	67.36 ± 0.14	256.0 ± 4.00
	300	68.13 ± 1.47	270.0 ± 3.0	73.84 ± 0.97	345.5 ± 11.50
	400	73.31 ± 2.47	348.0 ± 16.0	78.30 ± 0.61	475.0 ± 10.00
	500	77.71 ± 1.49	452.0 ± 6.0	81.98 ± 1.22	573.0 ± 4.00
175	0	48.08 ± 3.54	51.1 ± 1.6	46.14 ± 1.69	43.7 ± 0.70
	50	49.20 ± 1.20	56.7 ± 3.1	50.39 ± 0.39	70.1 ± 2.90
	100	52.58 ± 0.64	84.5 ± 0.75	55.94 ± 0.21	119.5 ± 1.50
	200	56.73 ± 1.61	123.0 ± 2.0	61.81 ± 1.31	194.0 ± 4.00
350	0	47.08 ± 2.92	49.7 ± 1.55	45.96 ± 0.65	47.4 ± 2.80
	50	45.60 ± 3.22	43.2 ± 0.55	46.67 ± 0.16	45.4 ± 0.40
	100	46.19 ± 0.73	45.9 ± 0.40	47.36 ± 1.02	53.5 ± 0.65
	200	47.74 ± 1.08	52.4 ± 0.70	50.61 ± 0.20	72.0 ± 2.65

The results obtained for flocculant B, in the presence of SPAM (Table 8), followed the same trend observed for flocculant A (Table 8), but showed slightly superior performance in terms of oil removal efficiency and aggregate formation. Increasing the flocculant concentration favored both parameters, especially in the absence of the demulsifier. Under these conditions, with 500 ppm of flocculant, efficiency of 92% and a D90 of 933.5 μm were achieved. With the addition of demulsifier, a reduction in efficiency was also observed, which was more pronounced in the combination of 350 ppm of demulsifier with 50 ppm of flocculant, where the efficiency dropped to 47% and the D90 was significantly reduced to 45.4 μm.

The results obtained for flocculants A and B indicated that the oil removal efficiency and the size of the aggregates formed were directly related to the flocculant concentration, in accordance with the bridging flocculation mechanism. Increasing the flocculant concentration favored greater interaction between the polymer chains and the colloidal particles, promoting adsorption and interconnection, resulting in larger aggregates (high D90). This aggregation occurred because the presence of more polymer chains reduces the electrostatic repulsion between the particles, facilitating sedimentation and maximizing oil removal. Conversely, the presence of the demulsifier hampered this mechanism, limiting aggregate formation and decreasing process efficiency, possibly due to saturation of the colloidal surfaces.

On the other hand, SPAM impaired the separation efficiency of the flocculants, probably due to the electrostatic shielding caused by the interaction between its sulfonate groups and the ions dissolved in the saline water, hindering coalescence, and also presenting a smaller particle size. Furthermore, the simultaneous presence of the demulsifier and SPAM limited the action of the flocculants, and this effect was more significant in the presence of 350 ppm of demulsifier.

### 3.9 Interfacial Tension Analysis

Interfacial tension measurements were performed to correlate the results with the efficiency of the flocculants obtained by jar testing. For this purpose, we conducted interfacial tension analyses of the following systems:

(1) Oil/water interface
(2) Oil/water interface with flocculant in the aqueous phase
(3) Oil/water interface with SPAM solubilized in brine
(4) Oil/water interface with demulsifier solubilized in oil
(5) Oil/water interface with SPAM solubilized in brine and demulsifier solubilized in oil.

Figure 7 shows the interfacial tension values as a function of the concentration of flocculants A and B, without SPAM and without demulsifier. The average efficiency of TOG reduction is included in the figure for comparison purposes. Initially, a reduction in interfacial tension was observed with increasing flocculant concentration, up to a certain concentration. For flocculant A, the interfacial tension decreased from 23.85 to 15.36 mN/m at a concentration of 200 ppm. This behavior is aligned with the principle of interfacial adsorption of macromolecules, where the presence of polymers at the interface reduces the interfacial energy.^53^ However, with 500 ppm of flocculant A, there was an increase in interfacial tension to 17.23 mN/m.

**Figure 7:**
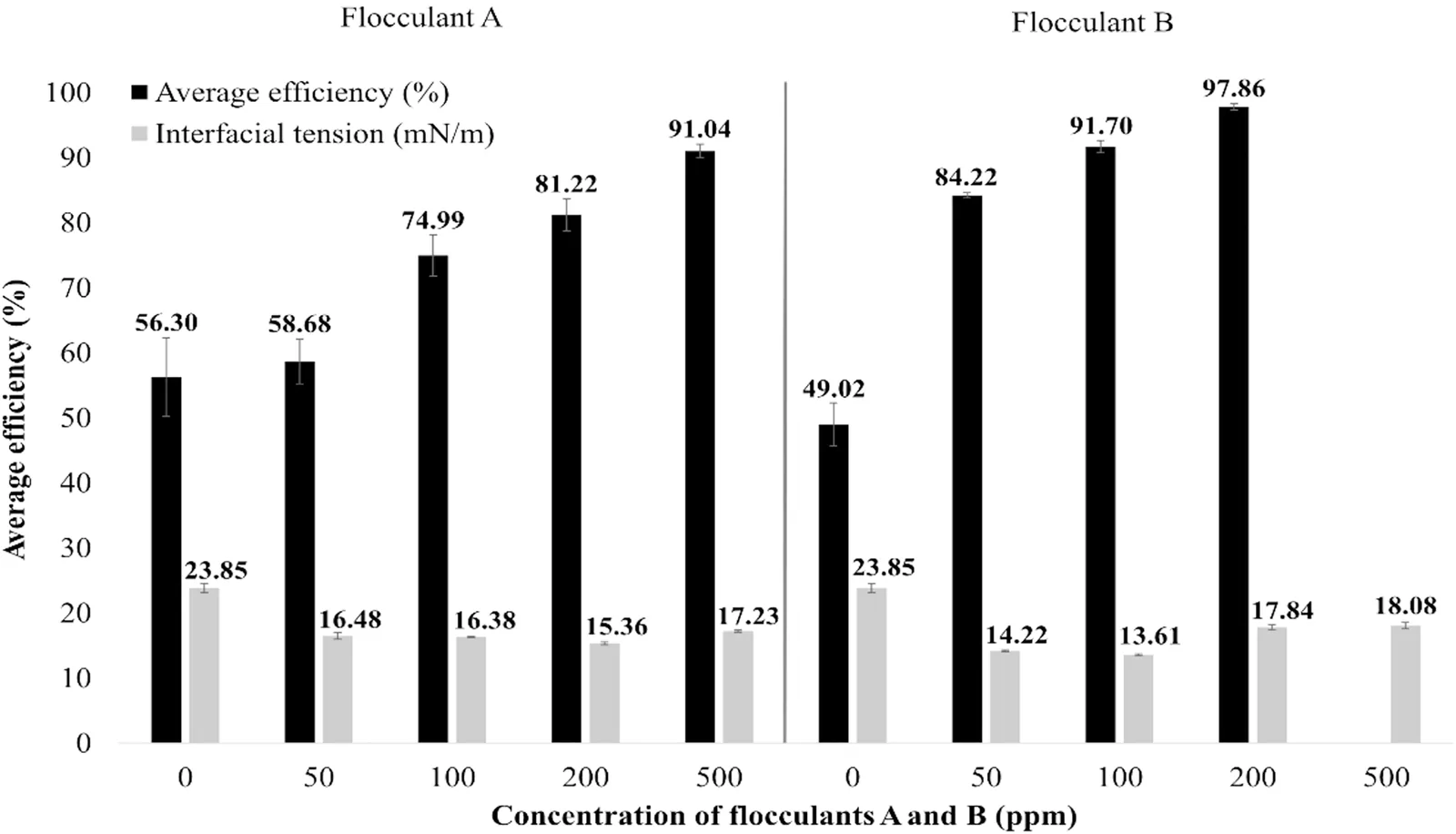
Correlation between the average TOG reduction efficiency and interfacial tension as a function of the concentration of flocculants A and B, without SPAM and without demulsifier.

For flocculant B, similar behavior was observed, but the reduction in interfacial tension occurred only up to 100 ppm, going from 23.85 mN/m (without flocculant) to 13.61 mN/m. From this concentration onward, there was an increase in interfacial tension, with values of 17.84 and 18.08 mN/m for 200 and 500 ppm of flocculant, respectively. This behavior suggests saturation of the interface, a phenomenon also observed for flocculant A. According to the literature,^53^ competition for adsorption sites or the occurrence of molecular rearrangements in the previously stabilized interface can cause disorganization of the interfacial layer, increasing the tension values.

The faster interface saturation observed for flocculant B compared to flocculant A can be attributed to distinct physicochemical characteristics between the two flocculants. Although both have low charge density, which favors the elongation of polymer chains by electrostatic repulsion and contributes to interfacial adsorption, flocculant B has an even lower charge density (0.22 mequiv/g) and possibly a higher molar mass. However, the hypothesis of a higher molar mass for flocculant B still requires experimental verification. This combination tends to generate longer and more flexible chains, which when adsorbed at the oil–water interface, occupy a larger interfacial area per molecule, promoting saturation at lower concentrations. The results of TOG reduction efficiency could only be compared to interfacial tension measurements up to the concentration where interface saturation did not occur for either flocculant.

The interfacial tension measurements in the presence of SPAM alone showed a reduction from 23.85 ± 0.72 mN/m to 19.23 ± 0.34 mN/m occurred. This reduction can be attributed to the amphiphilic nature of SPAM, which allows its adsorption at the oil–water interface, promoting a reduction in interfacial energy. The same behavior of interfacial tension reduction was observed in the presence of the demulsifier alone, where the tension declined from 23.85 ± 0.72 mN/m to 20.06 ± 0.58 mN/m. The reduction in interfacial tension in the presence of SPAM and the demulsifier separately may interfere with the efficiency of the flocculants, by hindering their migration to the interface. This behavior was confirmed when both SPAM and demulsifier were added together. The reduction in interfacial tension was even greater (from 23.85 ± 0.72 mN/m to 17.03 ± 0.08 mN/m).

Analyses of the flocculants in the presence of SPAM and the demulsifier, both separately and together, were not performed since saturation of the interface was observed with increasing flocculant concentration. Thus, the comparison between interfacial tension and TOG removal efficiency shows that a direct correlation between the reduction in interfacial tension and increased flocculation efficiency exists only at concentrations below saturation. Beyond saturation, interfacial tension ceases to be a reliable performance indicator, as the mechanism becomes governed by molecular rearrangements and the blocking of adsorption sites. The effects observed for SPAM and the demulsifier reinforce this relationship. Thus, the results indicate that flocculation efficiency depends not only on the reduction of interfacial tension but primarily on the availability of interfacial area for flocculant adsorption. Interfacial tension serves as a useful indicator only while the interface remains unsaturated; beyond this point, its interpretation must account for the effects of competition and interfacial reorganization

## 4 Conclusions

An efficient method was developed to prepare synthetic oily water using heavy/asphaltic crude oil, allowing us to evaluate how residual chemical additives affect the performance of polymeric flocculants applied in oil–water separation. We observed that the demulsifier, without the presence of flocculants did not alter the TOG at any of the concentrations, with and without SPAM. This result may be related to its low HLB value, which hinders the breaking of the O/W emulsion. In emulsions without SPAM, flocculant B outperformed flocculant A under almost all conditions. This result may be related to the difference in molar mass and lower charge density, favoring the formation of larger and more stable flocs. However, the hypothesis of a higher molar mass for flocculant B still requires experimental verification. The addition of the demulsifier alone in the presence of flocculants reduced their efficiency, with a more pronounced impact on flocculant A. The addition of SPAM alone reduced the efficiency of the flocculants at all oil concentrations used, with the impact being more pronounced when compared to that of the demulsifier. The combination of SPAM + demulsifier caused almost total suppression of the flocculants, demonstrating that the simultaneous presence of these additives strongly interferes with the flocculation process. Particle size analyses showed that higher concentrations of flocculant, without additives, formed larger aggregates, while in the presence of SPAM and demulsifier this formation was limited. Interfacial tension measurements showed an initial reduction in tension with increasing flocculant concentration, but there was saturation of the interface at the highest flocculant concentrations. When both SPAM and demulsifier were present, the reduction in interfacial tension was even greater, which may limit the area for flocculant action. In general, the presence of SPAM, both alone and in combination with the demulsifier, significantly compromised the efficiency of flocculants in the treatment of oily water. These results show the importance of considering the interactions between flocculants and chemical additives used in oil production to achieve more effective processes, thus allowing the selection of the best additive at the best concentration.
